# Supplementary material for: Can eating pleasure be a lever for healthy eating? A systematic scoping review of eating pleasure and its links with dietary behaviors and health
Source: PLoS One. 2020 Dec 21;15(12):e0244292. doi: 10.1371/journal.pone.0244292 (PMC7751982; doi:10.1371/journal.pone.0244292)
Supplement: S1 Table — (DOCX) [file pone.0244292.s001.docx]

**S1 Table.** Search strategies (other than Medline)

| Database: PsycInfo (OVID) |
| --- |
| 1. PLEASURE/ |
| 2. HEDONISM/ |
| 3. (pleasur* or pleasant* or fun or enjoy* or epicur* or hedon* or eudaimon* or eudaemon* or eudemon*).tw. |
| 4. 1 or 2 or 3 |
| 5. DIETS/ |
| 6. Eating Behavior/ |
| 7. Food Intake/ |
| 8. FOOD/ |
| 9. MEALTIMES/ |
| 10. NUTRITION/ |
| 11. Food Preferences/ |
| 12. Food Preparation/ |
| 13. Eating Attitudes/ |
| 14. (eat* or diet or diets or meal* or food or foods or nutrition).tw. |
| 15. 5 or 6 or 7 or 8 or 9 or 10 or 11 or 12 or 13 or 14 |
| 16. (4 and 15) not (Animals/ not Humans/) |
| 17. Limit 16 to (English or French) |

‘/ ’  indicates that a term is a subject heading (i.e., controlled vocabulary); ‘*’ represents truncation. For example, “pleasur*” finds terms that begin with the root term “pleasur”, such as pleasure, pleasures, pleasurable, etc. ‘.tw.’ is an alias for all of the fields in a database which contain text words and which are appropriate for a subject search.

| Database: ERIC (OVID) |
| --- |
| 1. (pleasur* or pleasant* or fun or enjoy* or epicur* or hedon* or eudaimon* or eudaemon* or eudemon*).tw. |
| 2. Dietetics/ |
| 3. Food/ |
| 4. Eating habits/ |
| 5. Lunch Programs/ or Breakfast Programs/ |
| 6. Nutrition/ |
| 7. (eat* or diet or diets or meal* or food or foods or nutrition).tw. |
| 8. 2 or 3 or 4 or 5 or 6 or 7 |
| 9. (1 and 8) not (Animals/ not Humans/) |
| 10. Limit 9 to (English or French) |

‘/ ’  indicates that a term is a subject heading (i.e., controlled vocabulary); ‘*’ represents truncation. For example, “pleasur*” finds terms that begin with the root term “pleasur”, such as pleasure, pleasures, pleasurable, etc. ‘.tw.’ is an alias for all of the fields in a database which contain text words and which are appropriate for a subject search.

| Database: Embase |
| --- |
| 1. 'pleasure'/de |
| 2. 'pleasantness'/de |
| 3. 'pleasure principle'/de |
| 4. 'enjoyment'/de |
| 5. 'hedonism'/de |
| 6. 'hedonic eating'/de |
| 7. pleasur*:ti,ab or pleasant*:ti,ab or fun:ti,ab or enjoy*:ti,ab or epicur*:ti,ab or hedon*:ti,ab or eudaimon*:ti,ab or eudaemon*:ti,ab or eudemon*:ti,ab |
| 8. #1 or #2 or #3 or #4 or #5 or #6 or #7 |
| 9. 'feeding behavior'/mj |
| 10. 'food preference'/mj |
| 11. 'meal'/mj |
| 12. 'diet'/mj |
| 13. 'eating habit'/mj |
| 14. 'eating'/mj |
| 15. 'food'/exp /mj |
| 16. 'food intake'/mj |
| 17. 'nutrition'/mj |
| 18. eat*:ti,ab or diet:ti,ab or diets:ti,ab or meal*:ti,ab or food:ti,ab or foods:ti,ab or nutrition:ti,ab |
| 19. #9 or #10 or #11 or #12 or #13 or #14 or #15 or #16 or #17 or #18 |
| 20. (#8 and #19) NOT ([animals]/lim NOT [humans]/lim) |
| 21. #20 AND ([english]/lim OR [french]/lim) |

‘/ ’  indicates that a term is a subject heading (i.e., controlled vocabulary); ‘de’: This option maps the search terms to the Emtree preferred indexing term; ‘exp’: This option maps the search term to the Emtree preferred indexing term and then searches for the related narrower or child terms; ‘mj’: This option maps the search terms to the Emtree preferred indexing term and then searches it as a major focus; ‘*’ represents truncation. For example, “pleasur*” finds terms that begin with the root term “pleasur”, such as pleasure, pleasures, pleasurable, etc. ‘ti,ab:’ is an alias for title/abstract.

| Database: Web of Science |
| --- |
| 1. (TS=((pleasur* OR enjoy* OR fun OR hedon* OR epicur* OR pleasant* OR eudaimon* OR eudaemon* OR eudemon*) AND (eat* OR food or foods OR diet OR diets OR meal* OR nutrition)) AND LANGUAGE: (English OR French) |

TS: Searches the topic fields in all databases. Topic fields include titles, abstracts, keywords and indexing fields such as systematics, taxonomic terms and descriptors; ‘*’ represents truncation. For example, “pleasur*” finds terms that begin with the root term “pleasur”, such as pleasure, pleasures, pleasurable, etc.

| Database: CINAHL (Interface EBSCOhost Research Databases) |
| --- |
| 1. (MH "Pleasure") |
| 2. TI pleasur* OR AB pleasur* |
| 3. TI enjoy* OR AB enjoy* |
| 4. TI fun OR AB fun |
| 5. TI hedon* OR AB hedon* |
| 6. TI epicur* OR AB epicur* |
| 7. TI pleasant* OR AB pleasant |
| 8. TI eudaimon* OR AB eudaimon* |
| 9. TI eudaemon * OR AB eudaemon* |
| 10. TI eudemon* OR AB eudemon* |
| 11. (MH "Diet") |
| 12. (MH "Meals") |
| 13. (MH "Eating Behavior") |
| 14. (MH "Eating") |
| 15. (MH "Food+") |
| 16. (MH "Food Habits") |
| 17. (MH "Food Preferences") |
| 18. (MH "Nutrition") |
| 19. (MH "Nutrition Policy") |
| 20. (MH "Public Health Nutrition") |
| 21. (MH "Nutrition Education") |
| 22. TI diets OR AB diets |
| 23. TI diet OR AB diet |
| 24. TI foods OR AB foods |
| 25. TI food OR AB food |
| 26. TI eat* OR AB eat* |
| 27. TI meal* OR AB meal* |
| 28. TI nutrition OR AB nutrition |
| 29. (S1 OR S2 OR S3 OR S4 OR S5 OR S6 OR S7 OR S8 OR S9 OR S10) AND (S11 OR S12 OR S13 OR S14 OR S15 OR S16 OR S17 OR S18 OR S19 OR S20 OR S21 OR S22 OR S23 OR S24 OR S25 OR S26 OR S27 OR S28) Limiters - Humain; Langue: English, French |

‘MH’ indicates that a term is a subject heading (i.e., controlled vocabulary); ‘+’: indicates that a subject heading is ‘exploded’ to include all of the narrower subject headings beneath it in the hierarchy; ‘*’ represents truncation. For example, “pleasur*” finds terms that begin with the root term “pleasur”, such as pleasure, pleasures, pleasurable, etc.; ‘TI’ is an alias for title; ‘AB’ is an alias for abstract.

| Database: ABI/INFORM Global (ProQuest) |
| --- |
| 1. (((ti(pleasur*) OR ab(pleasur*)) OR (ti(enjoy*) OR ab(enjoy*)) OR (ti(fun) OR ab(fun)) OR (ti(hedon*) OR ab(hedon*)) OR (ti(epicur*) OR ab(epicur*)) OR (ti(pleasant*) OR ab(pleasant*)) OR (ti(eudaimon*) OR ab(eudaimon*)) OR (ti(eudaemon*) OR ab(eudaemon*)) OR (ti(eudemon*) OR ab(eudemon*))) AND (MAINSUBJECT.EXACT("Diet") OR MAINSUBJECT.EXACT("Eating behavior") OR MAINSUBJECT.EXACT("Food") OR MAINSUBJECT.EXACT("Nutrition") OR MAINSUBJECT.EXACT("Meals") OR MAINSUBJECT.EXACT("Nutrition education") OR MAINSUBJECT.EXACT("Nutrition research") OR (ti(eat*) OR ab(eat*)) OR (ti(food) OR ab(food)) OR (ti(foods) OR ab(foods)) OR (ti(nutrition) OR ab(nutrition)) OR (ti(diet) OR ab(diet)) OR (ti(diets) OR ab(diets)) OR (ti(meal*) OR ab(meal*)))) AND la.exact("English" OR "French") AND stype.exact("Trade Journals" OR "Scholarly Journals" OR "Working Papers" OR "Reports" OR "Dissertations & Theses" OR "Other Sources" OR "Conference Papers & Proceedings") AND stype.exact(("Trade Journals" OR "Scholarly Journals" OR "Working Papers" OR "Reports" OR "Dissertations & Theses" OR "Other Sources" OR "Conference Papers & Proceedings") NOT ("Newspapers" OR "Magazines" OR "Wire Feeds")) |

‘MAINSUBJECT.EXACT’ indicates that a term is a subject heading (i.e., controlled vocabulary); ‘*’ represents truncation. For example, “pleasur*” finds terms that begin with the root term “pleasur”, such as pleasure, pleasures, pleasurable, etc.; ‘ti’ is an alias for title; ‘ab’ is an alias for abstract; ‘la.exact’ limits the search to specific language; ‘stype.exact’: limits the search to specific source types.

| Database: Sociological Abstracts (ProQuest) |
| --- |
| 1. ((MAINSUBJECT.EXACT("Diet") OR MAINSUBJECT.EXACT.EXPLODE("Food") OR MAINSUBJECT.EXACT("Feeding Practices") OR MAINSUBJECT.EXACT("Nutrition") OR ti(eat*) OR ab(eat*) OR ti(food) OR ab(food) OR ti(foods) OR ab(foods) OR ti(diet) OR ab(diet) OR ti(diets) OR ab(diets) OR ti(meal*) OR ab(meal*) OR ti(nutrition*) OR ab(nutrition*)) AND (ti(pleasur*) OR ab(pleasur*) OR ti(enjoy*) OR ab(enjoy*) OR ti(fun) OR ab(fun) OR ti(hedon*) OR ab(hedon*) OR ti(epicur*) OR ab(epicur*) OR ti(pleasant*) OR ab(pleasant*) OR ti(eudaimon*) OR ab(eudaimon*) OR ti(eudaemon*) OR ab(eudaemon*) OR ti(eudemon*) OR ab(eudemon*) OR MAINSUBJECT.EXACT("Hedonism"))) AND (stype.exact(("Scholarly Journals" OR "Dissertations & Theses") NOT ("Books" OR "Conference Papers & Proceedings" OR "Magazines" OR "Other Sources")) AND la.exact(("ENG" OR "FRE") NOT ("SPA" OR "POR" OR "GER" OR "ITA" OR "SLV" OR "HRV" OR "RUS" OR "SLA" OR "DUT" OR "POL" OR "CZE" OR "DAN" OR "FIN" OR "HUN" OR "JPN" OR "TUR"))) |

‘MAINSUBJECT.EXACT’ indicates that a term is a subject heading (i.e., controlled vocabulary); ‘MAINSUBJECT.EXACT.EXPLODE’: This option maps the search term and then searches for the related narrower or child terms; ‘*’ represents truncation. For example, “pleasur*” finds terms that begin with the root term “pleasur”, such as pleasure, pleasures, pleasurable, etc.; ‘ti’ is an alias for title; ‘ab’ is an alias for abstract; ; ‘la.exact’ limits the search to specific language; ‘stype.exact’: limits the search to specific source types.

| Database: ProQuest Dissertations & Theses Global (ProQuest) |
| --- |
| 1. ((((ab(pleasur*) OR ti(pleasur*)) AND la.exact("English" OR "French")) AND la.exact("English" OR "French")) OR (((ab(pleasant*) OR ti(pleasant*)) AND la.exact("English" OR "French")) AND la.exact("English" OR "French")) OR (((ab(hedon*) OR ti(hedon*)) AND la.exact("English" OR "French")) AND la.exact("English" OR "French")) OR (((ab(epicur*) OR ti(epicur*)) AND la.exact("English" OR "French")) AND la.exact("English" OR "French")) OR (((ab(fun) OR ti(fun)) AND la.exact("English" OR "French")) AND la.exact("English" OR "French")) OR (((ab(eudaimon*) OR ti(eudaimon*)) AND la.exact("English" OR "French")) AND la.exact("English" OR "French")) OR (((ab(eudaemon*) OR ti(eudaemon*)) AND la.exact("English" OR "French")) AND la.exact("English" OR "French")) OR (((ab(eudemon*) OR ti(eudemon*)) AND la.exact("English" OR "French")) AND la.exact("English" OR "French")) OR (((ab(enjoy*) OR ti(enjoy*)) AND la.exact("English" OR "French")) AND la.exact("English" OR "French"))) AND ((((ab(diet) OR ti(diet)) AND la.exact("English" OR "French")) AND la.exact("English" OR "French")) OR (((ab(diets) OR ti(diets)) AND la.exact("English" OR "French")) AND la.exact("English" OR "French")) OR (((ab(eat*) OR ti(eat*)) AND la.exact("English" OR "French")) AND la.exact("English" OR "French")) OR (((ab(meal*) OR ti(meal*)) AND la.exact("English" OR "French")) AND la.exact("English" OR "French")) OR (((ab(nutrition) OR ti(nutrition)) AND la.exact("English" OR "French")) AND la.exact("English" OR "French")) OR (((ab(food) OR ti(food)) AND la.exact("English" OR "French")) AND la.exact("English" OR "French")) OR (((ab(foods) OR ti(foods)) AND la.exact("English" OR "French")) AND la.exact("English" OR "French"))) |

‘*’ represents truncation. For example, “pleasur*” finds terms that begin with the root term “pleasur”, such as pleasure, pleasures, pleasurable, etc.; ‘ti’ is an alias for title; ‘ab’ is an alias for abstract; ; ‘la.exact’ limits the search to specific language.

**Google**

**Organization websites**

pleasure OR enjoyment OR enjoy OR hedonic OR pleasant OR fun OR eudaimonia OR epicurean AND eat OR food OR diet OR meal OR nutrition site:.org

plaisir OR épicurien OR hédoniste OR plaisant OR eudaimonia AND manger OR nourriture OR aliment OR alimentation OR repas OR nutrition site:.org

**Government of Australia**

pleasure OR enjoyment OR enjoy OR hedonic OR pleasant OR fun OR eudaimonia OR epicurean AND eat OR food OR diet OR meal OR nutrition (site:.gov.au)

**Government of Canada**

pleasure OR enjoyment OR enjoy OR hedonic OR pleasant OR fun OR eudaimonia OR epicurean AND eat OR food OR diet OR meal OR nutrition (site:.canada.ca OR site:.gc.ca)

plaisir OR épicurien OR hédoniste OR plaisant OR eudaimonia AND manger OR nourriture OR aliment OR alimentation OR repas OR nutrition (site:.canada.ca OR site:.gc.ca)

**Government of China**

pleasure OR enjoyment OR enjoy OR hedonic OR pleasant OR fun OR eudaimonia OR epicurean AND eat OR food OR diet OR meal OR nutrition (site:.gov.cn)

**Government of Denmark**

pleasure OR enjoyment OR enjoy OR hedonic OR pleasant OR fun OR eudaimonia OR epicurean AND eat OR food OR diet OR meal OR nutrition (site:.regeringen.dk)

**Government of Finland**

pleasure OR enjoyment OR enjoy OR hedonic OR pleasant OR fun OR eudaimonia OR epicurean AND eat OR food OR diet OR meal OR nutrition (site:.valtioneuvosto.fi)

**Government of France**

plaisir OR épicurien OR hédoniste OR plaisant OR eudaimonia AND manger OR nourriture OR aliment OR alimentation OR repas OR nutrition (site:.gouv.fr OR site:.gouvernement.fr)

**Government of Germany**

pleasure OR enjoyment OR enjoy OR hedonic OR pleasant OR fun OR eudaimonia OR epicurean AND eat OR food OR diet OR meal OR nutrition (site:.bundesregierung.de)

**Government of Greece**

pleasure OR enjoyment OR enjoy OR hedonic OR pleasant OR fun OR eudaimonia OR epicurean AND eat OR food OR diet OR meal OR nutrition (site:.gov.gr)

**Government of Ireland**

pleasure OR enjoyment OR enjoy OR hedonic OR pleasant OR fun OR eudaimonia OR epicurean AND eat OR food OR diet OR meal OR nutrition (site:.gov.ie)

**Government of Italy**

pleasure OR enjoyment OR enjoy OR hedonic OR pleasant OR fun OR eudaimonia OR epicurean AND eat OR food OR diet OR meal OR nutrition (site:[.governo.it](http://www.governo.it/))

**Government of Japan**

pleasure OR enjoyment OR enjoy OR hedonic OR pleasant OR fun OR eudaimonia OR epicurean AND eat OR food OR diet OR meal OR nutrition (site:.go.jp)

**Government of Malaysia**

pleasure OR enjoyment OR enjoy OR hedonic OR pleasant OR fun OR eudaimonia OR epicurean AND eat OR food OR diet OR meal OR nutrition (site:.gov.my)

**Government of Netherlands**

pleasure OR enjoyment OR enjoy OR hedonic OR pleasant OR fun OR eudaimonia OR epicurean AND eat OR food OR diet OR meal OR nutrition (site:.government.nl OR site:.overheid.nl)

**Government of United Kingdom**

pleasure OR enjoyment OR enjoy OR hedonic OR pleasant OR fun OR eudaimonia OR epicurean AND eat OR food OR diet OR meal OR nutrition (site:.gov.uk)

**Government of United States of America**

pleasure OR enjoyment OR enjoy OR hedonic OR pleasant OR fun OR eudaimonia OR epicurean AND eat OR food OR diet OR meal OR nutrition (site:.usa.gov)

**Government of Vietnam**

pleasure OR enjoyment OR enjoy OR hedonic OR pleasant OR fun OR eudaimonia OR epicurean AND eat OR food OR diet OR meal OR nutrition (site:.chinhphu.vn OR site:.gov.vn)
