# Supplementary material for: Can eating pleasure be a lever for healthy eating? A systematic scoping review of eating pleasure and its links with dietary behaviors and health
Source: PLoS One. 2020 Dec 21;15(12):e0244292. doi: 10.1371/journal.pone.0244292 (PMC7751982; doi:10.1371/journal.pone.0244292)
Supplement: S2 Table — (DOCX) [file pone.0244292.s002.docx]

| **S2 Table.** Characteristics of included documents | | | | | | | | | | | |  |
| --- | --- | --- | --- | --- | --- | --- | --- | --- | --- | --- | --- | --- |
| Source | Year of publication | Country | Publication type | Study/document design ^a^ | Journal discipline (Journal Citation Report) ^b^ | First author's discipline ^c^ | Target population ^d^ | Word(s) used to designate "pleasure" ^e^ |  | Research question(s) targeted by the document ^f^ | | |
|  |  |  |  |  |  |  |  |  |  | **Q1** | **Q2** | **Q3** |
| **Peer-reviewed articles** |  |  |  |  |  |  |  |  |  |  |  |  |
| Absolom & Roberts [110] | 2011 | UK | Peer-reviewed article | Qualitative | 1. Social sciences 2. Psychology 3. Rehabilitation | Rehabilitation | Adolescents | Enjoy* |  | ✓ |  |  |
| Adam et al. [160] | 2015 | Multi-countries | Peer-reviewed article | Intervention – prolonged exposure | 1. Nutrition  2. Medicine | Medicine | Young and middle-aged adults | Enjoy* |  |  | ✓ | ✓ |
| Ainuki et al. [29] | 2013 | Japan | Peer-reviewed article | Cross-sectional | 1. Public health 2. Nutrition | Social sciences | Young and middle-aged adults | Enjoy* |  | ✓ | ✓ |  |
| Andersen & Hyldig [71] | 2015 | Denmark | Peer-reviewed article | Qualitative | 1. Psychology 2. Nutrition | Food sciences | Young and middle-aged adults | Hedon* |  | ✓ |  |  |
| Appleton & McGowan [156] | 2006 | UK | Peer-reviewed article | Cross-sectional | 1. Psychology | Psychology | Young and middle-aged adults | Pleasur* |  |  | ✓ |  |
| Arch et al. [140] | 2016 | USA | Peer-reviewed article | Intervention – Single exposure | 1. Psychology | Psychology | Young and middle-aged adults | Enjoy* |  | ✓ | ✓ | ✓ |
| Bailly et al. [25] | 2015 | France | Peer-reviewed article | Cross-sectional | 1. Medicine | Psychology | Older adults | Pleasur* |  | ✓ | ✓ |  |
| Bissell et al. [133] | 2016 | UK | Peer-reviewed article | Qualitative | 1. Public health | Public health | Young and middle-aged adults | Pleasur* |  | ✓ |  |  |
| Cabanac [149] | 1971 | France | Peer-reviewed article | Intervention – single exposure | 1. Multidisciplinary sciences | Medicine | Young and middle-aged adults | Pleasur* |  | ✓ |  |  |
| Cachelin et al. [35] | 1998 | USA | Peer-reviewed article | Cross-sectional | 1. Medicine 2. Nutrition | Psychology | Young and middle-aged adults | Enjoy* |  |  | ✓ |  |
| Cornil & Chandon [21] | 2016 | USA | Peer-reviewed article | Cross-sectional | 1. Psychology 2. Nutrition | Business, Economy & Management | Young and middle-aged adults | Pleasur* AND Epicur* | | ✓ | ✓ |  |
| Cornil & Chandon [37] | 2016 | Multi-countries | Peer-reviewed article | Intervention – single exposure | 1. Business, Economy & Management | Business, Economy & Management | Children; young and middle-aged adults | Pleasur* |  | ✓ | ✓ | ✓ |
| Crawford et al. [28] | 2007 | Australia | Peer-reviewed article | Cross-sectional | 1. Public health 2. Nutrition | Nutrition / Physical activity | Young and middle-aged adults | Enjoy* |  | ✓ | ✓ |  |
| De Iulio & Kovacs [72] | 2014 | France | Peer-reviewed article | Qualitative | 1. Social sciences | Communication | Children | Pleasur* |  | ✓ |  |  |
| Ducrot et al. [36] | 2016 | France | Peer-reviewed article | Cross-sectional | 1. Nutrition | Nutrition | Young and middle-aged adults | Pleasur* |  | ✓ | ✓ |  |
| Ducrot et al. [27] | 2017 | France | Peer-reviewed article | Cross-sectional | 1. Nutrition | Nutrition | Young and middle-aged adults | Pleasur* |  | ✓ | ✓ |  |
| Dupuy et Poulain [65] | 2008 | France | Peer-reviewed article | Mixed-design | 1. Psychology | Social sciences | Children; adolescents; young and middle-aged adults | Plaisir |  | ✓ |  |  |
| Ensaff et al. [136] | 2016 | UK | Peer-reviewed article | Intervention – prolonged exposure | 1. Public health | Nutrition | Children | Enjoy* |  | ✓ | ✓ | ✓ |
| Gravel et al. [161] | 2014 | Canada | Peer-reviewed article | Intervention - prolonged exposure | 1. Nutrition | Nutrition | Young and middle-aged adults | Pleasur* |  |  | ✓ | ✓ |
| Gravel et al. [162] | 2014 | Canada | Peer-reviewed article | Intervention - prolonged exposure | 1. Food sciences | Nutrition | Young and middle-aged adults | Enjoy* |  |  | ✓ | ✓ |
| Guillemin et al. [44] | 2016 | France | Peer-reviewed article | Mixed-design | 1. Psychology 2. Nutrition | Patient-centred outcomes | Young and middle-aged adults | Pleasur* |  | ✓ |  |  |
| Hansen [127] | 2002 | Denmark | Peer-reviewed article | Intervention – single exposure | 1. Business, Economy & Management | Business, Economy & Management | Young and middle-aged adults | Pleasur* |  | ✓ |  |  |
| Hege et al. [159] | 2018 | Germany | Peer-reviewed article | Intervention - single exposure | 1. Psychology 2. Nutrition | Medicine | Young and middle-aged adults | Pleasur* |  |  | ✓ | ✓ |
| Hong et al. [141] | 2014 | USA | Peer-reviewed article | Intervention - single exposure | 1. Psychology | Psychology | Young and middle-aged adults | Enjoy* |  | ✓ | ✓ | ✓ |
| Huang & Wu [125] | 2016 | USA | Peer-reviewed article | Intervention - single exposure | 1. Food sciences | Business, Economy & Management | Young and middle-aged adults | Hedon* AND Enjoy* |  | ✓ | ✓ |  |
| Hunot et al. [157] | 2016 | UK | Peer-reviewed article | Cross-sectional | 1. Psychology 2. Nutrition | Public health | Young and middle-aged adults | Enjoy* |  |  | ✓ |  |
| Hur & Jang [151] | 2015 | USA | Peer-reviewed article | Cross-sectional | 1. Recreation | Business, Economy & Management / Recreation | Young and middle-aged adults | Pleasur* |  | ✓ | ✓ |  |
| Jackson et al. [145] | 2003 | USA | Peer-reviewed article | Cross-sectional | 1. Psychology | Medicine | Young and middle-aged adults | Pleasur* |  | ✓ | ✓ |  |
| Jallinoja et al. [8] | 2010 | Finland | Peer-reviewed article | Qualitative | 1. Public health 2. Social sciences | Public health | Young and middle-aged adults | Pleasur* |  | ✓ |  |  |
| Kauppinen-Raisanen et al. [67] | 2013 | Finland | Peer-reviewed article | Qualitative | 1. Food sciences | Business, Economy & Management | Young and middle-aged adults | Pleasur* |  | ✓ |  |  |
| Landry et al. [42] | 2018 | Canada | Peer-reviewed article | Qualitative | 1. Psychology 2. Nutrition | Nutrition | Young and middle-aged adults | Pleasur* |  | ✓ |  |  |
| Larson et al. [73] | 2014 | USA | Peer-reviewed article | Intervention – single exposure | 1. Business, Economy & Management 2. Psychology | Business, Economy & Management | Young and middle-aged adults | Enjoy* |  | ✓ |  |  |
| Lindeman & Stark [74] | 1999 | Finland | Peer-reviewed article | Cross-sectional | 1. Psychology 2. Nutrition | Psychology | Adolescents; young and middle-aged adults | Pleasur* |  | ✓ | ✓ |  |
| Lindeman & Stark [30] | 2000 | Finland | Peer-reviewed article | Cross-sectional | 1. Psychology 2. Nutrition | Psychology | Young and middle-aged adults | Pleasur* |  | ✓ | ✓ |  |
| Loh et al. [158] | 2013 | Malaysia | Peer-reviewed article | Cross-sectional | 1. Multidisciplinary sciences | Public health | Adolescents | Enjoy* |  |  | ✓ |  |
| Lupton [68] | 2000 | Australia | Peer-reviewed article | Qualitative | 1. Public health 2. Social sciences | Social sciences | Young and middle-aged adults | Pleasur* |  | ✓ |  |  |
| Macht et al. [43] | 2005 | Germany | Peer-reviewed article | Qualitative | 1. Psychology 2. Social sciences | Psychology | Young and middle-aged adults | Pleasur* AND Hedon* AND Enjoy* | | ✓ |  |  |
| Marquis & Shatenstein [75] | 2005 | Canada | Peer-reviewed article | Cross-sectional | 1. Nutrition | Nutrition | Young and middle-aged adults | Enjoy* |  | ✓ | ✓ |  |
| Marty et al. [26] | 2017 | France | Peer-reviewed article | Cross-sectional | 1. Nutrition 2. Medicine | Nutrition | Children | Pleasur* AND Hedon* | | ✓ | ✓ |  |
| Mason et al. [76] | 2017 | USA | Peer-reviewed article | Cross-sectional | 1. Psychology | Psychology | Young and middle-aged adults | Enjoy* |  | ✓ | ✓ |  |
| McClain et al. [150] | 2011 | USA | Peer-reviewed article | Cross-sectional | 1. Psychology | Medicine | Children | Pleasur* |  | ✓ | ✓ |  |
| Monnery-Patris et al. [128] | 2016 | France | Peer-reviewed article | Cross-sectional | 1. Psychology 2. Nutrition | Nutrition | Children | Pleasur* AND Hedon* | | ✓ |  |  |
| Moore [77] | 2013 | USA | Peer-reviewed article | Intervention - single exposure | 1. Business, Economy & Management | Business, Economy & Management / Physical activity | Young and middle-aged adults | Pleasur* AND Enjoy* | | ✓ |  |  |
| Newcombe et al. [78] | 2012 | Ireland | Peer-reviewed article | Qualitative | 1. Psychology 2. Nutrition | Business, Economy & Management | Older adults | Hedon* |  | ✓ |  |  |
| Niva [129] | 2007 | Finland | Peer-reviewed article | Qualitative | 1. Psychology 2. Nutrition | Business, Economy & Management | Young and middle-aged adults | Pleasur* |  | ✓ |  |  |
| Olsen & Tuu [33] | 2017 | Vietnam | Peer-reviewed article | Cross-sectional | 1. Food sciences | Business, Economy & Management | Adolescents | Hedon* |  | ✓ | ✓ |  |
| Onwezen & Bartels [79] | 2013 | Multi-countries | Peer-reviewed article | Cross-sectional | 1. Food sciences | Business, Economy & Management | Young and middle-aged adults | Enjoy* |  | ✓ |  |  |
| Otake & Kato [154] | 2017 | Japan | Peer-reviewed article | Cross-sectional | 1. Psychology 2. Social sciences | Psychology | Young and middle-aged adults | Pleasur* |  | ✓ |  |  |
| Petit et al. [7] | 2016 | France | Peer-reviewed article | Intervention - single exposure | 1. Multidisciplinary sciences | Business, Economy & Management | Young and middle-aged adults | Pleasur* |  | ✓ | ✓ | ✓ |
| Phan & Chambers [34] | 2016 | USA | Peer-reviewed article | Cross-sectional | 1. Food sciences | Food sciences | Young and middle-aged adults | Pleasur* |  | ✓ | ✓ |  |
| Phan & Chambers [13] | 2016 | USA | Peer-reviewed article | Cross-sectional | 1. Psychology 2. Nutrition | Food sciences | Young and middle-aged adults | Pleasur* |  | ✓ | ✓ |  |
| Prior & Limbert [111] | 2012 | UK | Peer-reviewed article | Mixed-design | 1. Medicine | Psychology | Adolescents | Enjoy* |  | ✓ | ✓ |  |
| Raghunathan et al. [15] | 2006 | USA | Peer-reviewed article | Intervention - single exposure | 1. Business, Economy & Management | Business, Economy & Management | Young and middle-aged adults | Hedon* AND Enjoy* |  | ✓ |  |  |
| Rappoport et al. [80] | 1992 | USA | Peer-reviewed article | Mixed-design | 1. Nutrition | Psychology | Young and middle-aged adults | Pleasur* |  | ✓ |  |  |
| Rappoport et al. [81] | 1993 | USA | Peer-reviewed article | Cross-sectional | 1. Psychology 2. Nutrition | Psychology | Young and middle-aged adults | Pleasur* |  | ✓ |  |  |
| Régnier [82] | 2014 | France | Peer-reviewed article | Qualitative | 1. Social sciences | Social sciences / Nutrition | Young and middle-aged adults | Plaisir |  | ✓ |  |  |
| Remick et al. [112] | 2009 | Canada | Peer-reviewed article | Cross-sectional | 1. Psychology | Psychology | Young and middle-aged adults | Pleasur* |  | ✓ | ✓ |  |
| Robinson et al. [147] | 2011 | UK | Peer-reviewed article | Intervention - single exposure | 1. Nutrition | Psychology | Young and middle-aged adults | Hedon* AND Enjoy* |  | ✓ | ✓ | ✓ |
| Robinson et al. [148] | 2012 | UK | Peer-reviewed article | Intervention - single exposure | 1. Nutrition | Psychology | Young and middle-aged adults | Enjoy* |  | ✓ | ✓ | ✓ |
| Roininen et al. [83] | 1999 | Finland | Peer-reviewed article | Intervention - single exposure | 1. Nutrition 2. Psychology | Food sciences | Young and middle-aged adults | Hedon* |  | ✓ |  |  |
| Roininen & Tuorila [84] | 1999 | Finland | Peer-reviewed article | Cross-sectional | 1. Food sciences | Food sciences | Young and middle-aged adults | Hedon* AND Pleasant* | | ✓ | ✓ |  |
| Roininen et al. [85] | 2000 | Finland | Peer-reviewed article | Qualitative | 1. Psychology 2. Nutrition | Food sciences | Young and middle-aged adults | Pleasur* AND Pleasant* | | ✓ |  |  |
| Roininen et al. [86] | 2001 | Multi-countries | Peer-reviewed article | Cross-sectional | 1. Psychology 2. Nutrition | Food sciences | Young and middle-aged adults | Pleasur* |  | ✓ | ✓ |  |
| Rozin et al. [69] | 1999 | Multi-countries | Peer-reviewed article | Cross-sectional | 1. Psychology 2. Nutrition | Psychology | Young and middle-aged adults | Pleasur* |  | ✓ |  |  |
| Rozin et al. [87] | 2003 | USA | Peer-reviewed article | Cross-sectional | 1. Psychology | Psychology | Young and middle-aged adults | Pleasur* |  | ✓ |  |  |
| Sasson et al. [32] | 2007 | USA | Peer-reviewed article | Intervention – prolonged exposure | 1. Nutrition | Nutrition | Young and middle-aged adults | Pleasur* |  |  | ✓ | ✓ |
| Smith & Hawks [126] | 2006 | USA | Peer-reviewed article | Cross-sectional | 1. Public health | Public health | Young and middle-aged adults | Pleasur* AND Enjoy* | | ✓ | ✓ |  |
| Somers et al. [88] | 2014 | Australia | Peer-reviewed article | Cross-sectional | 1. Nutrition 2. Medicine | Nutrition / Physical activity | Older adults | Pleasur* |  | ✓ | ✓ |  |
| Thogersen-Ntoumani et al. [146] | 2009 | Greece | Peer-reviewed article | Prospective | 1. Psychology | Physical activity | Adolescents | Pleasur* |  | ✓ | ✓ |  |
| Vinai et al. [152] | 2016 | Italy | Peer-reviewed article | Cross-sectional | 1. Psychology | Psychology | Young and middle-aged adults | Pleasur* |  | ✓ | ✓ |  |
| Vogel & Mol [142] | 2014 | Netherlands | Peer-reviewed article | Qualitative | 1. Public health 2. Social sciences | Social sciences | Young and middle-aged adults | Pleasur* AND Enjoy* | | ✓ |  |  |
| Wahl et al. [130] | 2017 | Germany | Peer-reviewed article | Cross-sectional | 1. Multidisciplinary sciences | Psychology | Young and middle-aged adults | Happiness |  | ✓ |  |  |
| Werle et al. [16] | 2013 | France | Peer-reviewed article | Intervention - single exposure | 1. Food sciences | Business, Economy & Management | Young and middle-aged adults | Pleasur* |  | ✓ |  |  |
| Yang et al. [137] | 2014 | Malaysia | Peer-reviewed article | Qualitative | 1. Business, Economy & Management 2. Recreation | Recreation | Young and middle-aged adults | Pleasur* AND Enjoy* | | ✓ | ✓ |  |
| Yang & Khoo-Lattimore [89] | 2015 | Taiwan | Peer-reviewed article | Qualitative | 1. Recreation | Business, Economy & Management / Recreation | Young and middle-aged adults | Enjoy* |  | ✓ | ✓ |  |
| **Theses/Dissertations** | |  |  |  |  |  |  |  |  |  |  |  |
| Baek [131] | 2009 | USA | Thesis/Dissertation | Intervention – single exposure | N/A | Recreation | Young and middle-aged adults | Hedon* |  | ✓ |  |  |
| Le bel [109] | 2000 | Canada | Thesis/Dissertation | Cross-sectional | N/A | Business, Economy & Management | Young and middle-aged adults | Hedon* AND Pleasant* | | ✓ | ✓ |  |
| Potestio [90] | 2012 | USA | Thesis/Dissertation | Mixed-design | N/A | Nutrition | Adolescents | Enjoy* |  | ✓ |  |  |
| Ray Chaudhury [91] | 2010 | USA | Thesis/Dissertation | Qualitative | N/A | Business, Economy & Management | Young and middle-aged adults | Pleasur* |  | ✓ |  |  |
| West [92] | 2003 | Canada | Thesis/Dissertation | Intervention - single exposure | N/A | Business, Economy & Management | Young and middle-aged adults | Pleasur* |  | ✓ |  |  |
| **Web pages** |  |  |  |  |  |  |  |  |  |  |  |  |
| Australian Government [143] | 2015 | Australia | Government website | Guidelines | N/A | N/A | All | Enjoy* |  | ✓ |  |  |
| Australian Government [132] | 2017 | Australia | Government website | Guidelines | N/A | N/A | All | Enjoy* |  | ✓ |  |  |
| Australian Government [97] | 2019 | Australia | Government website | Guidelines | N/A | N/A | All | Enjoy* |  | ✓ |  |  |
| Centre de recherche et d'information nutritionnelle (CERIN) [93] | 2018 | France | Organization website | Healthy eating promotion tool | N/A | N/A | All | Plaisir |  | ✓ |  |  |
| Goutons un monde meilleur [138] | 2011 | France | Organization website | Healthy eating promotion tool | N/A | N/A | All | Plaisir |  | ✓ |  |  |
| Gouvernement du Québec [105] | 2009 | Canada | Government website | Program description | N/A | N/A | Children | Plaisir |  | ✓ |  |  |
| Government of Brazil [23] | 2014 | Brazil | Government website | Guidelines | N/A | N/A | All | Pleasur* |  | ✓ |  |  |
| Government of Canada [107] | 2004 | Canada | Government website | Healthy eating promotion tool | N/A | N/A | Older adults | Pleasur* AND Fun AND Enjoy* |  | ✓ |  |  |
| Government of Canada [116] | 2007 | Canada | Government website | Guidelines | N/A | N/A | All | Plaisir |  | ✓ |  |  |
| Government of Canada [135] | 2010 | Canada | Government website | Report | N/A | N/A | Children; adolescents; young and middle-aged adults | Plaisir |  | ✓ |  |  |
| Government of Canada [94] | 2011 | Canada | Government website | Report | N/A | N/A | All | Plaisir |  | ✓ |  |  |
| Government of Canada [117] | 2019 | Canada | Government website | Guidelines | N/A | N/A | All | Enjoy* |  | ✓ |  |  |
| Government of Canada [155] | 2019 | Canada | Government website | Guidelines | N/A | N/A | All | Enjoy* |  | ✓ |  |  |
| Government of Canada [115] | 2019 | Canada | Government website | Guidelines | N/A | N/A | All | Enjoy* |  | ✓ |  |  |
| Government of Canada [22] | 2019 | Canada | Government website | Guidelines | N/A | N/A | All | Enjoy* |  | ✓ |  |  |
| Government of Canada [144] | 2019 | Canada | Government website | Guidelines | N/A | N/A | All | Enjoy* |  | ✓ |  |  |
| Government of Canada [117] | 2019 | Canada | Government website | Guidelines | N/A | N/A | All | Enjoy* |  | ✓ |  |  |
| Government of South Australia [113] | 2009 | Australia | Government website | Healthy eating promotion tool | N/A | N/A | Children | Fun |  | ✓ |  |  |
| Harvard School of Public Health Prevention Research Center (HPRC) [122] | 2011 | USA | Organization website | Healthy eating promotion tool | N/A | N/A | Children; young and middle-aged adults | Fun |  | ✓ |  |  |
| Institut national de la recherche agronomique (INRA) [106] | 2010 | France | Organization website | Report | N/A | N/A | All | Plaisir |  | ✓ |  |  |
| République Française [121] | 2002 | France | Government website | Guidelines | N/A | N/A | All | Plaisir |  | ✓ |  |  |
| République Française [102] | 2005 | France | Government website | Program description | N/A | N/A | All | Plaisir |  | ✓ |  |  |
| République Française [100] | 2010 | France | Government website | Program description | N/A | N/A | Children; adolescents; young and middle-aged adults | Plaisir |  | ✓ |  |  |
| République Française [104] | 2010 | France | Government website | Report | N/A | N/A | All | Plaisir |  | ✓ |  |  |
| République Française [118] | 2011 | France | Government website | Program description | N/A | N/A | Children | Plaisir |  | ✓ |  |  |
| République Française [134] | 2012 | France | Government website | Report | N/A | N/A | All | Plaisir |  | ✓ |  |  |
| République Française [103] | 2013 | France | Government website | Program description | N/A | N/A | Children; adolescents | Plaisir |  | ✓ |  |  |
| République Française [99] | 2014 | France | Government website | Report | N/A | N/A | All | Plaisir |  | ✓ |  |  |
| République Française [119] | 2016 | France | Government website | Report | N/A | N/A | All | Plaisir |  | ✓ |  |  |
| République Française [120] | 2016 | France | Government website | Report | N/A | N/A | All | Plaisir |  | ✓ |  |  |
| République Française [101] | 2018 | France | Government website | Report | N/A | N/A | Children; adolescents | Plaisir |  | ✓ |  |  |
| République Française [153] | 2019 | France | Government website | Program description | N/A | N/A | Children; adolescents | Plaisir |  | ✓ |  |  |
| République Française [139] | 2019 | France | Government website | Guidelines | N/A | N/A | Young and middle-aged adults | Plaisir |  | ✓ |  |  |
| U.S. Government [124] | 2016 | USA | Government website | Healthy eating promotion tool | N/A | N/A | Children | Fun |  | ✓ |  |  |
| ¸  U.S. Government [108] | 2016 | USA | Government website | Healthy eating promotion tool | N/A | N/A | All | Enjoy* |  | ✓ |  |  |
| U.S. Government [123] | 2019 | USA | Government website | Healthy eating promotion tool | N/A | N/A | Older adults | Pleasur* AND Enjoy* |  | ✓ |  |  |
| Victoria State Government [98] | 2012 | Australia | Government website | Healthy eating promotion tool | N/A | N/A | Older adults | Pleasur* AND Fun AND Enjoy* |  | ✓ |  |  |
| Victoria State Government [96] | 2018 | Australia | Government website | Healthy eating promotion tool | N/A | N/A | All | Enjoy* |  | ✓ |  |  |
| Victoria State Government [95] | 2018 | Australia | Government website | Healthy eating promotion tool | N/A | N/A | Children; adolescents | Enjoy* |  | ✓ |  |  |

^a^ Single exposure indicates that participants were exposed to a treatment on only one occasion (acute exposure) while prolonged exposure indicates that participants were exposed to treatment(s) on several occasions (i.e., longer-term interventions).

^b^ The journal name was used to find the associated discipline using Journal Citation Reports (InCites, 2018). When the journal was not indexed in this database, the scope of the journal was used to identify the discipline. See S2 Table for the description of each discipline. Theses, dissertations and web pages were excluded from this analysis. Q. 1: n=66; Q. 2, n=44 and Q. 3, n=13.

^c^ The first author’s affiliation was used to determine the author’s discipline. See S2 Table for the description of each field. Websites were excluded from this analysis. Q. 1: n=71; Q. 2, n=45 and Q. 3, n=13.

^d^ For articles, theses and dissertations, the target population was children when the mean age was between 5 and 12 years, adolescents when the mean age was between 12 and 18 years, young and middle-aged adults when the mean age was between 18 and 65 years and older adults when the mean age was over 65 years. When no mean age was provided, the age range was used to identify the target population the most represented. For web pages, we used the target population identified through the document by the authors. The target population was considered as “all” when the document targeted all strata of the population or when no target population was identified by authors.

^e^ For articles, theses and dissertations, words identified as designating pleasure were those found in the title/abstract. For web pages, words identified were those found in the entire document.

^f^ Research question 1: How is eating pleasure conceptualized (i.e., key dimensions) in scientific research and organization/government documents?; Research question 2: What is the current available scientific evidence about associations of eating pleasure, and its key dimensions, with dietary behaviors and health outcomes?; Research question 3: What are the most promising strategies using eating pleasure to promote healthy dietary behaviors identified through intervention studies?
