# Supplementary material for: Can eating pleasure be a lever for healthy eating? A systematic scoping review of eating pleasure and its links with dietary behaviors and health
Source: PLoS One. 2020 Dec 21;15(12):e0244292. doi: 10.1371/journal.pone.0244292 (PMC7751982; doi:10.1371/journal.pone.0244292)
Supplement: S3 Table — (DOCX) [file pone.0244292.s003.docx]

**S3 Table.** Description of disciplines

| **Discipline** | **Description** |
| --- | --- |
| Business, Economy & Management | Covers resources concerned with all aspects of business and the business world. These may include marketing and advertising, forecasting, planning, administration, organizational studies, compensation, strategy, retailing, consumer research, economy, and management. Covers resources relating to business history and business ethics. Also covered are resources on management science, organization studies, strategic planning and decision-making methods, leadership studies, and total quality management. |
| Communication | Covers resources on the study of the verbal and non-verbal exchange of ideas and information. Included here are communication theory, practice and policy, media studies (journalism, broadcasting, advertising, etc.), mass communication, public opinion, speech, business and technical writing as well as public relations. |
| Food sciences | Covers resources concerning various aspects of food research and production, including food additives and contaminants, food chemistry and biochemistry, meat science, food microbiology and technology, dairy science, food engineering and processing, cereal science, brewing, and food quality and safety. |
| Medicine | Covers resources dealing with the diagnosis and treatment (general medicine, internal medicine, clinical physiology, pain management, military and hospital medicine, pharmacology and pharmacy) of different conditions and diseases. Also concerns resources studying normal functioning of living cells, tissues and human systems (cardiac, respiratory, endocrinology, etc.). |
| Multidisciplinary sciences | Covers resources of a very broad or general character in sciences (physics, chemistry, mathematics, biology, sociology, etc.). |
| Nutrition | Covers resources concerning many aspects of nutrition, including general nutrition, nutrition and metabolism, nutrition science, clinical nutrition, vitamin research and nutritional biochemistry. Dietetics, the application of nutritional principles, is also included in this category. |
| Patient-centred outcomes | Covers resources concerning scientific approaches in the patient-centred outcomes field that encourage exchanges between academics, pharmaceutical companies, and international organisations around the world in the service of incorporating the patient’s voice into every step of the development process of new treatments. |
| Physical activity | Covers resources concerning all aspects of physical activity, ranging from: children to older people, elite athletes to sedentary adults, and healthy participants to those recovering from injury or living with chronic diseases. |
| Psychology | Covers resources on the study of human behavior and mental processes. It also includes fields of clinical treatment and psychiatry that study mental health and behavioral disorders. |
| Public health | Covers resources on health services, health care management, health policy and planning, health economics, health behavior, and health education. |
| Recreation | Covers resources that focus on all aspects of recreation and leisure studies, sport, hospitality, and travel and tourism. |
| Rehabilitation | Rehabilitation covers resources on therapy to aid in the recovery or enhancement of physical, cognitive, or social abilities diminished by birth defect, disease, injury, or aging. |
| Social sciences | Covers resources that focus on the study of human society, social structures, and social change as well as human behavior as it is shaped by social forces. Covers resources relating to the scientific study of human beings: anthropology, history, philosophy, sociology, archaeology and geography. Also, includes resources on occupation and the political and social effects of biomedical research. |
