# Supplementary material for: Can eating pleasure be a lever for healthy eating? A systematic scoping review of eating pleasure and its links with dietary behaviors and health
Source: PLoS One. 2020 Dec 21;15(12):e0244292. doi: 10.1371/journal.pone.0244292 (PMC7751982; doi:10.1371/journal.pone.0244292)
Supplement: S4 Table — (DOCX) [file pone.0244292.s004.docx]

| **S4 Table.** Key dimensions of eating pleasure, with description of sub-dimensions | | | | |  |
| --- | --- | --- | --- | --- | --- |
| Dimensions | Number of records  n=110  n (%) | Description | | References |  |
| Sensory experiences | 56 (50.9) | To experience food / meals with the five senses, i.e., to experience its sensory qualities. | | [7,8,15,21-23,25,26,30,33,37,42-44,65,67,68,71-109] |  |
| Taste | 48 (43.6) | To experience the taste of the food. Taste includes the five basic tastes, i.e., sweet, salty, sour, bitter, umami. | | [7,8,15,21-23,25,26,30,33,37,42-44,65,68,71-80,82-93,96,98-102,104,106-108] |  |
| Appearance | 18 (16.4) | To experience the appearance of the food (e.g., shape, color, presentation). | | [21,23,33,43,44,71,72,75,86,91,93-96,98,100,102,105] |  |
| Texture | 14 (12.7) | To experience the texture of the food when touching it or eating it. | | [21,23,37,42,43,68,71,72,91,93-95,100,108] |  |
| General sensory aspects | 13 (11.8) | To experience the sensory attributes of the food in general. | | [37,42,71,80,81,85,92,94,97,102,103,105,109] |  |
| Smell | 12 (10.9) | To experience the smell of food. Includes also the retro-olfaction when eating the food. | | [21,23,33,37,43,72,75,77,91,100,102,105] |  |
| Aesthetic | 5 (4.5) | To experience the "beauty" of the food, an interpretation related to aesthetics (keyword: Aesthetic). | | [21,42,67,80,81] |  |
| Sound | 4 (3.6) | To experience the noise or sound of the food before or during consumption. | | [37,93,100,105] |  |
| Temperature | 1 (0.9) | To experience the temperature of the food. | | [68] |  |
| Social experiences | 47 (42.7) | To integrate social experiences into the act of eating. | | [22,23,27,29,36,42-44,65,67-69,71,72,75,78,80,81,91,92,95,98-104,106,107,110-113,115-126] |  |
| Eating with others | 36 (32.7) | To eat with other people and/or share the food / meal. | | [22,23,27,29,36,42-44,65,67,68,71,72,75,78,92,95,98-100,102,103,106,107,110-113,115-119,121,123] |  |
| Preparing meals with others | 13 (11.8) | To prepare meals / to cook with others. | | [22,23,42,43,67,68,91,98,100,113,118,120,124] |  |
| Respecting shared norms and practices | 13 (11.8) | To respect the system of norms, values and practices specific to a social space. | | [22,42-44,65,67,68,71,72,80,91,99,117] |  |
| Culture & Traditions | 10 (9.1) | To respect the rules, norms, practices specific to the culture/social environment in which you live (e.g., family, local and national traditions). | | [22,42,65,67,68,71,72,80,99,117] |  |
| Social rituals | 3 (2.7) | To appropriate practices/performances around the act of eating in a social context (e.g., taking a glass of wine before the guests arrive, putting on beautiful clothes when hosting or going out to eat). | | [43,44,68] |  |
| Citizen responsibility | 2 (1.8) | To respond to one’s civic responsibilities (keywords: world solidarity, duty). | | [72,91] |  |
| Identity claim | 1 (0.9) | To assert oneself by rejecting or discriminating against products and to appropriate experience. | | [65] |  |
| General social experiences | 7 (6.4) | To respond to the social experience as a whole. | | [69,80,81,92,101,125,126] |  |
| Receiving and serving people | 4 (3.6) | To receive people at home for a meal and to serve them food (keywords: hosting, having people over). | | [44,67,104,122] |  |
| Serving food that our loved ones like | 4 (3.6) | To choose or prepare food that is appreciated by others. | | [27,36,75,102] |  |
| Eating alone | 3 (2.7) | To eat alone. | | [43,44,65] |  |
| Knowing the person who produces / prepares the food / meal | 3 (2.7) | To know the people who grow or produce the food or prepare the meal consumed. | | [22,91,118] |  |
| Letting yourself be served | 2 (1.8) | To accept invitations from friends or family and/or to let others cook and wash dishes. | | [67,68] |  |
| Food characteristics | 30 (27.3) | To eat food with certain attributes, other than those related to sensory ones. | | [8,15,16,27,36,42-44,65,67,68,71,75,78,80-82,85,91,92,97,104,119,121,127-132] |  |
| Healthy | 10 (9.1) | To eat food that is perceived or labelled as "healthy". | | [15,16,42,43,67,68,71,82,85,129] |  |
| Types of food | 7 (6.4) | To eat specific food products. | | [65,78,80,81,92,128,130] |  |
| Unhealthy | 6 (5.5) | To eat food that is perceived or labelled as "unhealthy". | | [8,15,71,85,97,132] |  |
| Fresh | 4 (3.6) | To eat food perceived as fresh. | | [42,44,67,68] |  |
| Convenient / simple | 3 (2.7) | To eat food that is easy and convenient to eat. | | [42,67,68] |  |
| Local | 3 (2.7) | To eat food that is grown locally. | | [44,104,119] |  |
| Natural | 3 (2.7) | To eat food that is not processed. | | [43,85,91] |  |
| Satiating | 3 (2.7) | To eat food that is satiating (filling). | | [42,43,121] |  |
| Special | 3 (2.7) | To eat food considered special (e.g., luxurious, sophisticated, not eaten often) | | [42,80,127] |  |
| Well-known | 3 (2.7) | To eat food that is well-known. | | [42,75,131] |  |
| Good nutritional value | 2 (1.8) | To eat food because of its nutrient content (e.g., proteins, fats, carbohydrates, fibers). | | [42,67] |  |
| Original | 2 (1.8) | To eat food perceived as original. | | [27,36] |  |
| Well-prepared | 2 (1.8) | To eat food for which the preparation has been well done. | | [43,67] |  |
| Adequate energy content | 1 (0.9) | To eat food with an energy content appropriate to your needs. | | [43] |  |
| Authentic | 1 (0.9) | To eat food perceived or labelled as "authentic". | | [67] |  |
| Homemade | 1 (0.9) | To eat homemade foods or food labelled as "homemade". | | [67] |  |
| Organic | 1 (0.9) | To eat food that is perceived or labelled as "organic". | | [44] |  |
| Food preparation process | 21 (19.1) | To experience the steps involved in buying and preparing food before eating it. | | [22,23,28,42-44,68,72,91,92,100,104,113,118-120,122,133-136] |  |
| Cooking | 19 (17.3) | To prepare/cook food to make a meal or snack. | | [22,23,28,42-44,68,72,92,100,104,113,118,120,122,133-136] |  |
| Gardening | 5 (4.5) | To have a garden and grow food to eat afterwards. | | [22,72,91,113,118] |  |
| Grocery shopping | 5 (4.5) | To do food shopping (excludes the farmers' market). | | [22,28,44,119,133] |  |
| Going to the farmers' market | 2 (1.8) | To go to the farmer's market. | | [42,91] |  |
| Novelty | 18 (16.4) | To learn something about food or to experiment with something new or unusual in terms of taste, foods or meals. | | [22,27,33,36,42,44,67,68,71,72,89,92,104,107,110,113,122,137] |  |
| Discovering new foods, dishes and tastes | 14 (12.7) | To discover new foods, new dishes or new tastes. | | [22,27,36,42,44,67,68,71,72,89,107,110,122,137] |  |
| Learning about food | 6 (5.5) | To learn about nutrition, food and dishes. | | [71,72,92,104,113,122] |  |
| Breaking the routine | 2 (1.8) | To change your habits or break the routine. | | [33,44] |  |
| Variety | 16 (14.5) | To consume a diet whose components are varied. | | [22,23,27,36,42-44,71,96,99,122,124,132,137-139] |  |
| Variety in foods | 9 (8.2) | To consume a variety of different foods. | | [23,42,44,71,96,99,132,137,139] |  |
| Variety in flavours | 6 (5.5) | To consume a variety of food with different flavors. | | [22,42-44,71,138] |  |
| Variety in the way food is prepared | 5 (4.5) | To consume a varied menu in terms of how food is prepared. Originality and creativity in the preparation. | | [27,36,42,122,124] |  |
| Mindful eating | 15 (13.6) | To consume food while savoring every bite; being aware of the present moment and not being distracted. | | [23,43,44,96,97,108,116-118,139-144] |  |
| Visceral eating | 14 (12.7) | To satisfy short-term visceral impulses triggered by hunger, external cues or internal emotional cues. | | [8,13,21,34,42-44,68,79,85,92,107,145,146] |  |
| Rewarding yourself | 10 (9.1) | To use food to congratulate yourself, giving yourself the right to eat a certain food as a reward because you deserve it (keywords: treat, reward, indulge). | | [8,13,34,42,68,79,85,107,145,146] |  |
| Coping with emotions | 5 (4.5) | To use food to escape or help yourself through negative emotions or to manage stress. | | [21,42-44,92] |  |
| Eating impulsively, based on appeal | 1 (0.9) | To eat on impulse, wanting to have something, inability to resist. | | [44] |  |
| Disinhibition | 1 (0.9) | To eat a large amount of food without being able to stop. | | [42] |  |
| Eating in response to external cues | 1 (0.9) | To eat food in response to stimuli external to the person. | | [21] |  |
| Place | 13 (11.8) | To consume food in a specific environment or place. | | [42-44,65,67-69,92,98,99,107,110,126] |  |
| Eating at restaurant | 11 (10.0) | To eat out (e.g., restaurants). | | [42-44,65,67,68,92,98,99,107,110] |  |
| Eating while travelling / tourism | 5 (4.5) | To eat while travelling, as tourists. | | [42,65,67,69,126] |  |
| Eating at home | 3 (2.7) | To eat at home. | | [43,65,68] |  |
| Eating outdoors | 2 (1.8) | To eat outdoors, in nature, on a terrace, etc. | | [42,43] |  |
| Eating at work / school | 1 (0.9) | To eat at work or school. | | [65] |  |
| Eating in front of the television | 1 (0.9) | To eat in front of the television. | | [42] |  |
| Memories | 12 (10.9) | To consider food / meals through memorable food experiences from the past. | | [42,43,67-69,72,89,112,125,126,147,148] |  |
| Atmosphere | 11 (10.0) | To benefit from an atmosphere created by lighting, music, ambient temperature and decoration, among other things, while eating a food / meal. | | [22,23,43,44,67,68,99,107,118,127,142] |  |
| Psychological / physical state during food intake | 11 (10.0) | To experience a psychological and physical well-being state when eating. | | [33,43,80,81,85,90,92,137,149-151] |  |
| Experiencing emotions | 9 (8.2) | To experience emotions when eating. | | [33,43,80,81,85,90,92,137,150] |  |
| Experiencing a physical well-being state in general | 2 (1.8) | To experience a general physical well-being state when eating. | | [90,151] |  |
| Experiencing a sense of relaxation | 2 (1.8) | To experience a sense of relaxation when eating. | | [43,137] |  |
| Experiencing a sense of satisfaction | 2 (1.8) | To experience a sense of satisfaction when eating. | | [43,151] |  |
| Alliesthesia state | 1 (0.9) | To respond to the interdependence between one’s internal state and the perceived pleasure or displeasure of stimuli. The internal state is constantly changing, and any stimulus that can help correct an error or need will be perceived as pleasant. | | [149] |  |
| Food anticipation | 10 (9.1) | To anticipate the consumption of a food / meal. | | [43,69,71,77,92,112,125,126,151,152] |  |
| Special occasions | 10 (9.1) | To consume food on special occasions (e.g., holidays, birthday parties, weekends) | | [8,42,65,67,68,71,79,98,144,153] |  |
| Having the choice | 10 (9.1) | To feel free to choose food or the place where food is eaten | | [8,42-44,99,110,113,119,121,153] |  |
| Food intake structure | 9 (8.2) | To structure food intake throughout the day according to one’s preferences and in a balanced way. | | [28,42-44,71,72,78,99,130] |  |
| Diet structure | 5 (4.5) | To structure meals and snacks throughout the day as one’s preferences. | | [28,44,71,99,130] |  |
| Balanced diet | 4 (3.6) | To consume a balanced diet. | | [42,44,71,72] |  |
| Meal composition | 3 (2.7) | To put food on the plate according to one’s preferences (i.e., the proportion of food and where it is placed on the plate). | | [43,71,78] |  |
| Meal structure | 1 (0.9) | To structure the meal according to one’s preferences (e.g., order of courses, duration of the meal). | | [43] |  |
| Portion size | 1 (0.9) | To receive the size of portion one prefers. | | [78] |  |
| Taking time | 9 (8.2) | To take time buying, preparing and consuming food, to eat in a relaxed environment. | | [42-44,65,68,104,121,137,153] |  |
| Health considerations | 8 (7.3) | To make food choices and develop eating habits that help maintain good health. | | [8,22,42,71,78,82,90,129] |  |
| Balancing pleasure and health | 6 (5.5) | To allow oneself both healthy and unhealthy foods to balance health and pleasure in one’s diet, to permit oneself unhealthy food after eating healthy food. | | [8,42,71,78,82,129] |  |
| Making healthy choices | 5 (4.5) | To consider that one has made a good choice for one’s health. | | [22,71,82,90,129] |  |
| Restraining food intake | 2 (1.8) | To control food intake and weight through restrictive food choices. | | [42,82] |  |
| Food preferences | 8 (7.3) | To choose or eat the food one prefers, to respect one’s food preferences. | | [27,36,68,71,81,82,154,155] |  |
| Psychological / physical state after food intake | 7 (6.4) | To experience a psychological and physical well-being state after eating a meal / snack. | | [42-44,71,75,137,138] |  |
| Feeling full | 4 (3.6) | To feel full after eating a meal / snack. | | [42-44,71] |  |
| Feeling relax | 2 (1.8) | To feel relaxed after eating a meal / snack. | | [43,137] |  |
| Feeling satisfied | 2 (1.8) | To feel satisfied after eating a meal / snack. | | [43,44] |  |
| Having energy | 2 (1.8) | To feel one has enough energy after food intake to carry out activities. | | [71,75] |  |
| Feeling as meeting his/her body needs | 2 (1.8) | To feel that the needs of the body are fulfilled after eating a meal / snack. | | [137,138] |  |
| Feeling light | 1 (0.9) | To feel light, not too heavy after eating a meal / snack. | | [42] |  |
| Eating habits | 4 (3.6) | To choose or eat certain foods in accordance with eating habits, i.e., to eat food that the person is used to eating. | | [8,43,65,71] |  |
| Ideological considerations | 4 (3.6) | To choose and eat food considering the ideological dimensions one values, including its political, intellectual and spiritual dimensions. | | [44,91,92,104] |  |
| Environmental movement | 2 (1.8) | To choose food because it has a lower negative impact on the environment. | | [44,91] |  |
| General intellectual consideration | 2 (1.8) | To choose or eat food that is in keeping with one’s general intellectual values. | | [92,104] |  |
| Personal growth | 1 (0.9) | To choose to a diet whose consequences stimulate spiritual and mental growth. | | [92] |  |
|  |  |  |  | | |
